# Genetic delimitation of *Oreocharis* species from Hainan Island

**DOI:** 10.3897/phytokeys..32427

**Published:** 2020-08-26

**Authors:** Shao-Jun Ling, Xin-Ting Qin, Xi-Qiang Song, Li-Na Zhang, Ming-Xun Ren

**Affiliations:** 1 Key Laboratory of Genetics and Germplasm Innovation of Tropical Special Forest Trees and Ornamental Plants (Hainan University), Ministry of Education, Haikou 570228, China Hainan University Haikou China; 2 Center for Terrestrial Biodiversity of the South China Sea, Hainan University, Haikou 570228, China Hainan University Haikou China

**Keywords:** genetic differentiation, genetic diversity, morphological similarity, *
Oreocharis
*

## Abstract

Hainan Island harbours an extraordinary diversity of Gesneriaceae with 14 genera and 23 species, amongst which two species and one variety are recognised in the genus *Oreocharis*. These three *Oreocharis* taxa are all Hainan-endemics and show a complex geographical distribution pattern with considerable morphological intermixtures. In this study, we combined DNA (nuclear ITS sequences and cpDNA*trn*L-*trn*F and *ycf*1b) to evaluate genetic delimitation for 12 *Oreocharis* populations from the island, together with morphological similarity analysis using 16 morphological traits. The results showed Hainan *Oreocharis* taxa were monophyletic with relative low genetic diversity within populations, highly significant genetic differentiation amongst populations and a significant phylogeographical structure. The 12 populations formed three genetically distinct groups, roughly correspondent to the currently recognised two species and one unknown lineage. The PCA analyses of morphological traits indicate three distinctive groups, differing mainly in petal colour and corolla shapes. The roles of river and mountain isolations in the origin and distribution of these three lineages are discussed.

## Introduction

Hainan Island is the largest tropical island in China, with an area of 33,920 km^2^. As a biodiversity hotspot in the world (Myers et al. 2000), Hainan Island has a species-rich and remarkable endemic flora (Francisco-Ortega et al. 2010), which is remarkably richer in endemic genus than Taiwan Island (36,193 km^2^) and contains almost twice the number of Gesneriaceae species than Sri Lanka (65,610 km^2^), which is twice the size of Hainan Island (Ranasinghe et al. 2019). The richest biodiversity is concentrated in the south-central mountains of the island (Li and Wang 2005; Xing 2012; Yang 2013), such as Mt. Wuzhi (the highest peak with 1867 m) and Mt. Yingge (1812 m). Xing (2012) and Ling et al. (2017) identified 14 genera and 23 species of Gesneriaceae on Hainan Island, amongst which two genera (*Metapetrocosmea* W.T. Wang and *Cathayanthe* Chun) and eight species (including one variety) are endemic (Ling et al. 2017; Jiang et al. 2017).

Interestingly, there are three recognised taxa of *Oreocharis* Bentham on Hainan Island (*O. flavida* Merrill, *O. dasyantha* Chun and O. dasyantha Chun var. ferruginosa Pan) and all are endemic to the island (Wei 2010; Xing 2012; Yang 2013; Ling et al. 2017). During three years’ observation, we found these *Oreocharis* taxa to possess diverse floral syndromes in a single currently recognised species and mixed distribution of different species (Wei 2010; Xing 2012; Yang 2013), together with considerable genetic differentiations amongst populations (Xing et al. 2018). The presence of a variety, i.e. O. dasyantha var. ferruginosa, further complicates the taxonomy classification of Hainan *Oreocharis*.

Here, we sampled 12 populations of *Oreocharis* taxa covering its entire distribution range on Hainan Island and examined their molecular phylogenetic relationships with one nuclear DNA fragment and two combined chloroplast DNA sequences separately. We also quantitatively analysed 16 morphological traits with principal component analysis (PCA). We aim to determine (1) whether or not the currently recognised three species or variety can be supported by genetic data (2) what factors (e.g. geographic isolation, pollination isolation, climate or intrinsic traits) are responsible for the evolution and maintenance of these Hainan-endemic *Oreocharis*?

## Materials and methods

### Materials collection and DNA extraction

**Figure 1.**
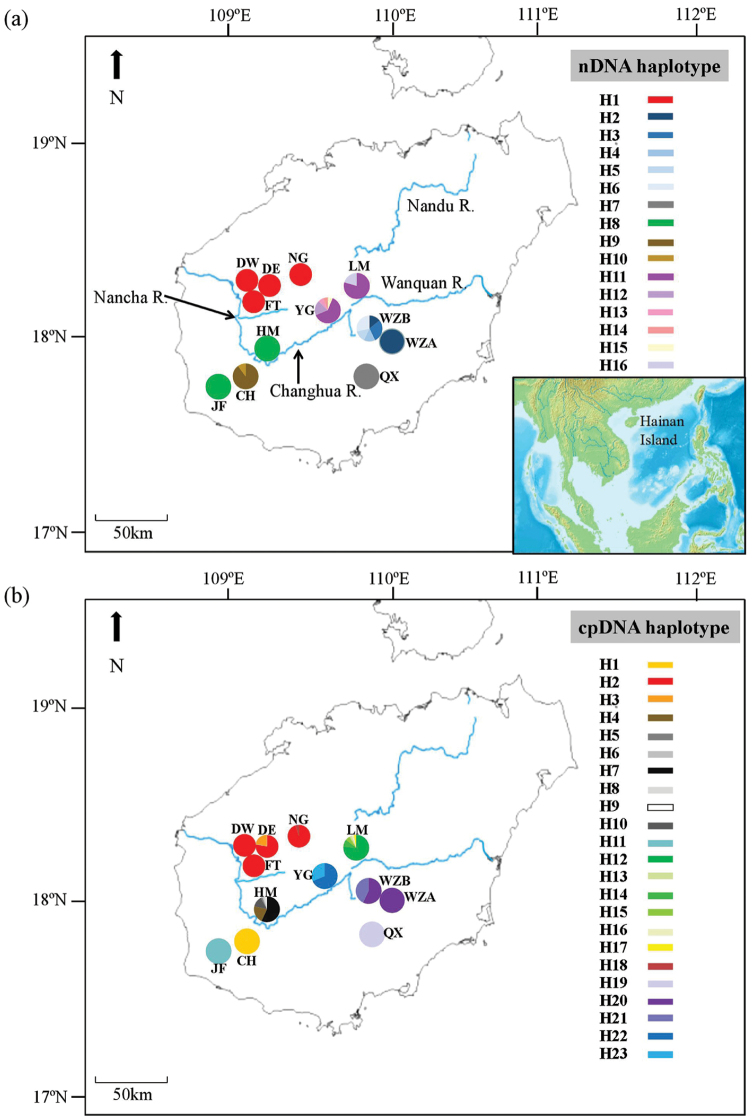
Sampling sites and nucleotype and haplotype distribution of nuclear ITS (**a**) and cpDNA*trn*L-F and *ycf*1b (**b**) of *Oreocharis* lineages in Hainan Island.

Twelve geographic populations of *Oreocharis* taxa covering all the suitable habitats of the genus on the island were collected, including populations DW (Dongwu in Bawangling), DE (Donger in Bawangling), FT (Futou in Bawangling), NG (Nangao), HM (Mt. Houmi), JF (Mt. Jianfeng) and CH (Chahe at the foot of Mt. Jianfeng) from *O. dasyantha*, populations QX (Mt. Qixian), WZA (Wuzhi A in Mt. Wuzhi) and WZB (Wuzhi B in Mt. Wuzhi) from *O. flavida* and populations YG (Mt. Yingge) and LM (Mt. Limu) from unidentified *Oreocharis* sp. (Table 1, Fig. 1). Fresh leaves were collected from the south-central mountains in Hainan Island in 2015, 2016 and 2017 and dry stored in silica gel. In total, 238 leaf samples from 12 populations that represented the whole geographical range of *Oreocharis* taxa on Hainan Island were collected (Fig. 1).

**Table 1.** Sampled populations and nucleotypes/haplotypes information calculated from nrDNA and cpDNA of 12 *Oreocharis* populations. Private nucleotypes/haplotypes (nucleotype/haplotype occurs in only one population) are given in Bold.

Putative populations	Sampling site	Population	Longitude/ Latitude	Sampling size	Elevation (m)	ITS			*trn*L-F and *ycf*1b		
						Haplotype (No. of individuals)	Hd	Pi × 10^3^	Haplotype (No. of individuals)	Hd	Pi × 10^3^
*O. dasyantha*	Dongwu in Mt. Bawang	DW	109°41'52"/ 18°53'55"	17	1163	H1(17)	0	0	H2(17)	0	0
	Donger in Mt. Bawang	DE	109°10'27"/ 19°05'07"	9	1011	H1(9)	0	0	H2(7), **H3(2)**	0.389	0.75
	Mt. Futou	FT	109°41'01"/ 18°53'51"	11	1200	H1(11)	0	0	H2(11)	0	0
	Mt. Nangao	NG	109°19'06"/ 19°10'48"	19	1350	H1(19)	0	0	H2(18), **H18(1)**	0.105	0.07
O. dasyantha var. ferruginosa	Mt. Houmi	HM	109°08'44"/ 18°53'50"	51	1400	H8(51)	0	0	**H4(14)**, **H5(1)**, **H6(1)**, **H7(29)**, **H8(1)**, **H9(1)**, **H10(7)**	0.622	0.52
	Mt. Jianfeng	JF	108°52'43"/ 18°43'10"	15	1100	H8(15)	0	0	**H11(15)**	0	0
	Riverside at Chahe	CH	108°59'00"/ 18°44'30"	10	300	**H9(9)**, **H10(1)**	0.2	0.31	**H1(10)**	0	0
*O. flavida*	Mt. Qixian	QX	109°42'16"/ 18°42'41"	17	1100	**H7(17)**	0	0	**H19(17)**	0	0
	Mt. Wuzhi	WZA	109°41'52"/ 18°53'55"	32	1800	H2(32)	0	0	H20(32)	0	0
	Mt. Wuzhi	WZB	109°41'29"/ 18°54'19"	7	1058	H2(1), **H3(2)**, **H4(1)**, **H5(1)**, **H6(2)**	0.905	2.51	H20(4), **H21(3)**	0.571	0.37
*Oreocharis* sp.	Mt. Yingge	YG	109°33'06"/ 19°02'21"	16	1249	**H11(1)**, H12(10), **H13(3)**, **H14(1)**, **H15(1)**	0.6	1.92	**H22(11)**, **H23(5)**	0.458	0.29
	Mt. Limu	LM	109°44'44"/ 19°10'10"	34	1350	H12(27), **H16(7)**	0.337	0.52	**H12(26)**, **H13(1)**,**H14(3)**, **H15(2)**, **H16(1)**, **H17(1)**	0.414	0.49
Sum				238			2.042	5.26		2.559	2.49

Total genomic DNA for each individual was extracted using CTAB methods (Doyle and Doyle 1987) and served as a template for the polymerase chain reaction. AL2000 DNA marker (Aidlab Biotechnologies Co. Ltd) was used to detect DNA quality and quantity on 0.8% agarose gels stained with 2.5 μl Goldview (Aidlab Biotechnologies Co. Ltd) in DTU-48 spectrophotometer (Hangzhou Miu Instruments Co. Ltd, China).

### PCR amplification and sequencing

One nuclear ribosomal DNA (nrDNA) sequence, the ITS region comprising spacer 1, the 5.8S gene and spacer 2 (White et al. 1990) and two chloroplast DNA (cpDNA) intron–spacer region *trn*L-*trn*F (Taberlet et al. 1991) and *ycf*1b (Dong et al. 2015) were used in this study (Table 2). PCR reactions were set up in a volume of 25 μl consisting of 20 μl ddH_2_O, 2.5 μl 10×Buffer, 0.5 μl dNTPs (10 mM), 0.5 μl each 5 μM primer, 0.5 μl DNA template and 0.5 μl 5 U/μl Taq polymerase (Aidlab Biotechnologies Co. Ltd). PCR was conducted in a 2720 Thermal cycler (Applied Biosystems by Life Technologies, made in Singapore) and Veriti 96-Well Thermal Cycler (Applied Biosystems by Life Technologies, made in Singapore). The PCR programme for nrDNA and *trn*L-*trn*F was designed for an initial denaturation at 94 °C 5 min, followed by 35 cycles of 1 min at 94 °C, 1 min at 55 °C, 1 min at 72 °C and with a final extension of 10 min at 72 °C. Amplification of *ycf*1b used the following protocol: 4 min at 94 °C, 35 cycles of 30 s at 94 °C, 40 s at 58 °C and 1 min at 72 °C, ending with 10 min at 72 °C. All the PCR products were checked by electrophoresis. Then purification and sequencing of PCR products were finally sequenced by an ABI 3730 DNA Analyzer based on the BigDye Terminator Cycle Sequencing Ready Kit (Applied Biosystems, Foster City, CA) in BGI (Beijing Genomics Institution), the chemistry and primers being used above in BGI.

**Table 2.** Primers used for DNA amplification of *Oreocharis* taxa and genetic diversity. S, polymorphic sites, h, number of haplotypes, Hd, haplotypes diversity, π, nucleotide diversity, K, average number of nucleotide difference.

DNA fragment	Primers sequences	S	h	Hd	π	K	Fragment size	Tajima’s *D*	Fu’s *Fs*	Reference
ITS	ITS4: 5’TCCTCCGCTTATTGATATGC 3’	56	16	0.820	0.02178	14.067	670 bp	1.53380	17.662***	White et al. 1990
	ITS5 HP: 5’GGAAGGAGAAGTCGTAACAAGG 3’									
*trn*L-F	c: 5’CGAAATCGGTAGC GCTACG 3’	16	11	0.805	0.00428	3.460	843 bp	0.77872	2.529	Taberlet 1991
	f: 5’ATTTGAACTGGTGA CACGAG 3’									
*ycf*1b	*ycf1*bF: 5’ACATATG CCAAAGTGATGGAAAA 3’	29	12	0.871	0.01243	8.890	725 bp	2.05208	12.821***	Dong et al. 2015
	*ycf1*bR: 5’CCTCGCCGAAAATCTGATTGTTGTGAAT 3’									
*trn*L-F and *ycf*1b		55	23	0.887	0.00845	13.214	1568 bp	1.33642	8.426*	

### The systematic position of Oreocharis taxa in Hainan Island

In order to explore the systematic position of *Oreocharis* taxa in Hainan Island, we followed Möller et al. (2011a, b) and Chen et al. (2014) and used 57 other *Oreocharis* species with suitable DNA sequences in the study. Finally, a total of 60 species were included in the phylogenetic analysis. We manually aligned all sequences using MEGA v.6.5 (Kumar et al. 2008) and excluded ambiguous positions from the alignments. The two no-coding gene ITS1/2 and *trn*L-*trn*F were concatenated to a single matrix by the programme SequenceMatrix v.1.7.8 (Vaidya et al. 2011) after a congruency test by PAUP* 4.0a164 (Swofford 2003). We inferred the optimal model of nucleotide substitution by MRMODELTEST 2.3 (Nylander 2004), based on the AIC (Akaike Information Criteria) (Akaike 1981). In addition, the most suitable model GTR+I+G was used in both ML and BI analysis. Maximum Likelihood (ML) analysis was conducted using MEGA v.6.5 (Kumar et al. 2008) with the optimal substitution models to carry out 1000 bootstrap (BS) replicates. Bayesian Inference (BI) analysis was conducted using MrBayes version 3.1.2 (Huelsenbeck and Ronquist 2001). The Markov Chain Monte Carlo (MCMC) was analysed for 10 million generations and sampling every 10000 generations for two independent Bayesian runs. The first 2500 trees (25% of total trees) were discarded as burn-in and the remaining trees were summarised in a 50% majority-rule consensus tree with the posterior probabilities (PP). The mean and posterior of each branch were visualised by FIGTREE v.1.4.2 (Rambaut 2009). Sequences used are showed in Appendix 2.

### Genetic diversity and differentiation

The original chromatograms from both directions of the ITS and cpDNA sequences obtained were evaluated with the software BioEdit (Hall 1999) for base confirmation and contiguous sequences editing, then sequences were manually aligned, where necessary, using MEGA v.6.5 (Kumar et al. 2008) and ambiguous positions were excluded from the alignments. All sequences have been deposited in GenBank (MK587942–MK588003). Subsequently, we combined the two no-coding cpDNA regions as a single locus by the programme SequenceMatrix v.1.7.8 (Vaidya et al. 2011). Then, we performed a Partition Homogeneity Test based on the combined cpDNA and an Incongruence Length Difference Test, based on combined ITS and cpDNA using PAUP* v. 4.0a164 (Swofford 2003).

The number of nucleotypes/haplotypes, number of nucleotypes/polymorphic sites (S), nucleotype/haplotype diversity (*h*), nucleotide diversity (π) and measures of DNA divergence (K) values were analysed by the programme DNASP v. 6.12.01 (Rozas et al. 2017) for each population and Fu’s *Fs* (Fu 1997) and Tajima’s *D* (Tajima 1989) values were tested for vital deviations from the null hypothesis of neutral evolution and constant population size, based on the ITS and cpDNA sequences separately. We generated the geographical distribution of nucleotypes/haplotypes according to sampling information (Table 1).

Genetic diversity within populations (*Hs*; Nei 1973), in total populations (*H_T_*), total gene diversity index (*N_ST_*) and genetic differentiation index within populations (*G_ST_*) were measured using Haplonst (Pons and Petit 1996) and *G_ST_* and *N_ST_* compared by the U test (Pons and Petit 1996) based on the ITS and cpDNA sequences separately.

The Analysis of Molecular Variance (AMOVA) was conducted to estimate genetic variation which was assigned within and amongst populations using GENALEX v. 6.503 (Peakall and Smouse 2012), based on the ITS and cpDNA sequences separately.

### Phylogenetic relationships

Phylogenetic relationships of nucleotypes/haplotypes were inferred with BI using MrBayes v. 3.2.6 (Ronquist et al. 2012). According to test above, *O. sinohenryi*, which had the closest phylogenetic relationships with the Hainan *Oreocharis* taxa, were used as outgroups with sequences of nrDNA.

Prior to Bayesian analysis, the optimal model of nucleotide substitution was detected for each gene using MRMODELTEST v. 2.3 (Nylander 2004), based on the AIC (Akaike 1981). Two independent Bayesian runs of MCMC were performed for 10 million generations, sampling every 10000 generations. We accessed the Chain convergence in Tracer v. 1.7.1 (Rambaut and Drummond 2007) by checking the effective sample size (ESS) that was larger than 200 for each parameter. To further explore the relationships amongst unique nucleotypes, genealogical relationships were inferred from Median-Joining network (MJ) of NETWORK v. 5.001 (http://www.fluxus-Engineering.com/).

### Neighbour-joining (NJ) tree and structure

All sequences of each population were chosen to represent effective geographic populations themselves. The method for the Neighbour-joining (NJ) tree was selected to build the phylogenetic relationship of *Oreocharis* taxa populations in Hainan Island by MEGA v.6.5, with Kimura two-parameter model (Kimura 1980), based on the ITS and two combined cpDNA sequences separately.

A Bayesian clustering approach conducted in STRUCTURE v. 2.3.4 (Earl and Vonholdt 2012) was used to detect the population genetic structure of the Hainan *Oreocharis* taxa, based on ITS and combined cpDNA sequences separately. The number of possible clusters (*K*) was set from 1 to 10 and each *K* run 10 times. Each run comprised a burn-in period of 1 × 10^5^ interactions with 1 × 10^5^MCMC steps after burning. The most suitable value of *K* was determined from Structure Harvester (Pritchard et al. 2000; http://taylor0.biology.ucla.edu/structureHarvester/) by using Δ*K* and the log-likelihood value. Finally, the result from programme STUCTURE for the best *K* value was drawn in CLUMPAK server (Jakobsson and Rosenberg 2007; http://clumpak.tau.ac.il/index.html).

### Isolation by distance (IBD)

To detect whether there was local genetic variation under geographically limited dispersal, isolation by distance (IBD) for each population was tested by a Mantel test in GENALEX between pairwise genetic distance (uncorrected sequence divergence (Dxy) for nuclear DNA and cpDNA) and geographical distance.

### Morphological traits

To characterise phenotype diversity and differences amongst populations, we measured and observed 16 morphological characters, including floral and leaf traits of at least 30 individuals for each population. The measured floral traits were, (i) corolla colour (yellow tube with orange lip, yellow, orange), (ii) corolla shape and type (tubular, thin tubular, campanulate), (iii) corolla width (< 1.49 cm, 1.5 cm - 1.99 cm, > 2.0 cm), (iv) corolla mouth width (< 0.5 cm, > 0.5 cm), (v) floral tube length (< 0.99 cm, 1 cm - 1.49 cm, > 1.5 cm), (vi) sepal length (short, long) and (vii) number of petals (five, six).

Five stamen traits were included in the analyses: (i) anther position (included-anthers hidden inside the floral tube, floral throat-anthers lying in the throat of floral tube, exerted-anthers are exposed outside the floral tube), (ii) stamen type (monomorphic, didynamous), (iii) pollen presentation (simultaneous, separately for each pair), (iv) anther shape (oval, horseshoe) and (v) hair on filament (absent, present).

Two stigma characters and two leaf traits were also included in the analyses: (i) location of stigma (included-stigma hidden inside the floral tube, throat-stigma lying in the throat of floral tube, exerted-stigma is exposed outside the floral tube), (ii) number of stigma (one, two), (iii) serration of leaf edge (present, absent) and (iv) leaf epidermal hair in abaxial side (absent, present). Measurements were taken with a rectilinear scale and rounded to the nearest 0.1 mm.

Principal Component Analysis (PCA) was conducted in SPSS v. 19.0 (Liu and Li 2014) to determine the traits with the highest value for classification and the plotting map.

## Results

### Monophyly of the Hainan Oreocharis taxa

The combined ITS1/2 and *trn*L-F datasets of Hainan *Oreocharis* taxa with other 57 *Oreocharis* species were 568 and 871 bp long, amongst which 233 and 89 were polymorphic sites and 141 and 38 were parsimony informative sites, respectively. The aligned dataset was 1439 bp long with a total number of 305 polymorphic sites measured, of which 160 were parsimony informative sites. There was no significant incongruence, based on the incongruence length difference (ILD) test between the ITS1/2 and *trn*L-F (p > 0.05).

Both the BI and ML analysis showed Hainan *Oreocharis* taxa being monophyly with PP (posterior probability) = 0.79 and BS (bootstrap value) = 38% (Appendix 1). In addition, *O. sinohenryi*, whose regions are restricted in South China (Guangxi and Guangdong Province), is the sister to Hainan *Oroecharis* taxa in the current tree with relatively high support (Appendix 1).

### Genetic diversity and differentiation

The aligned ITS sequence matrix comprised in total of 670 basepairs (bp). A total number of 56 polymorphic sites were present, of which 48 were parsimony-informative, which allowed the identification of 16 different nucleotypes from a size of 238 samples (Table 1, Fig. 1a). Four nucleotypes, H1, H2, H8 and H12, were shared amongst populations, the other 12 nucleotypes were private, i.e. present in only one population (Table 1, Fig. 1a).

The combined alignment of the two cpDNA regions was in total 1615 bp long (858 and 757 bp for *trn*L-*trn*F and *ycf*1b, respectively) with a significant rate of homogeneity (*P* = 1) in the congruency test, indicating that there was no significant difference in the laboratory between the two cpDNA regions. The alignment contained 55 polymorphic sites and 8 indels (Table 2). A total of 23 chloroplast haplotypes were present amongst the 238 samples (Fig. 1b, Table 1). Of these, only two haplotypes, H2 and H20, were shared amongst several populations, whereas the other 21 haplotypes were private (Table 1, Fig. 1b). The combined dataset, based on ITS and cpDNA as pairwise ILD tests, showed that the two DNA regions were not significantly different from each other (*P* > 0.05).

Haplotypes diversity (Hd) and nucleotide diversity (Pi) for each population are summarized in Table 1 and there is little difference between nrDNA and cpDNA. Generally, except for population WZB, YG and LM presented high genetic diversity in both nrDNA and cpDNA, nuclear gene ITS in population CH and chloroplast gene *trn*L-F and *ycf*1b in population DE, NG and HM showed variable genetic diversity, the rest of the populations having very low nucleotide and haplotype diversity (Table 1).

In total, the average intrapopulation diversity *H*_S_ was lower than the genetic diversity *H*_T._ Both in ITS and cpDNA sequences, total gene diversity index (*N*_ST_) was not significantly greater than the genetic differentiation index within populations (*G*_ST_, P > 0.05), revealing that Hainan *Oreocharis* taxa have no correspondence between haplotype comparability and geographic distribution (Appendix 3).

The AMOVA indicated that almost all variation (99% and 97%) was partitioned amongst populations, which was higher than the variation (1% and 3%) within populations, based on the ITS and cpDNA data, respectively, revealing highly significant genetic differentiation amongst populations (Table 3).

**Table 3.** Genetic diversity and Analysis of Molecular Variance (AMOVA) based on the ITS and combined *trn*L-F and *ycf*1b sequences in *Oreocharis* taxa.

DNA fragment	*H_S_*	*H_T_*	*G_ST_*	*N_ST_*	Source of variation	d.f.	Sum of squares	Variance components	Percentage of variation (%)	*F*_ST_	r
ITS	0.170	0.884	0.808	0.982	Amongst populations	11	2353.579	213.962	11.176	99%	
					Within populations	226	28.698	0.127	0.127	1%	2382.277
cpDNA	0.215	0.913	0.765	0.977	Amongst populations	11	2497.917	227.083	11.848	97%	
					Within populations	226	88.752	0.393	0.393	3%	2586.668

### Phylogenetic relationship

**Figure 2.**
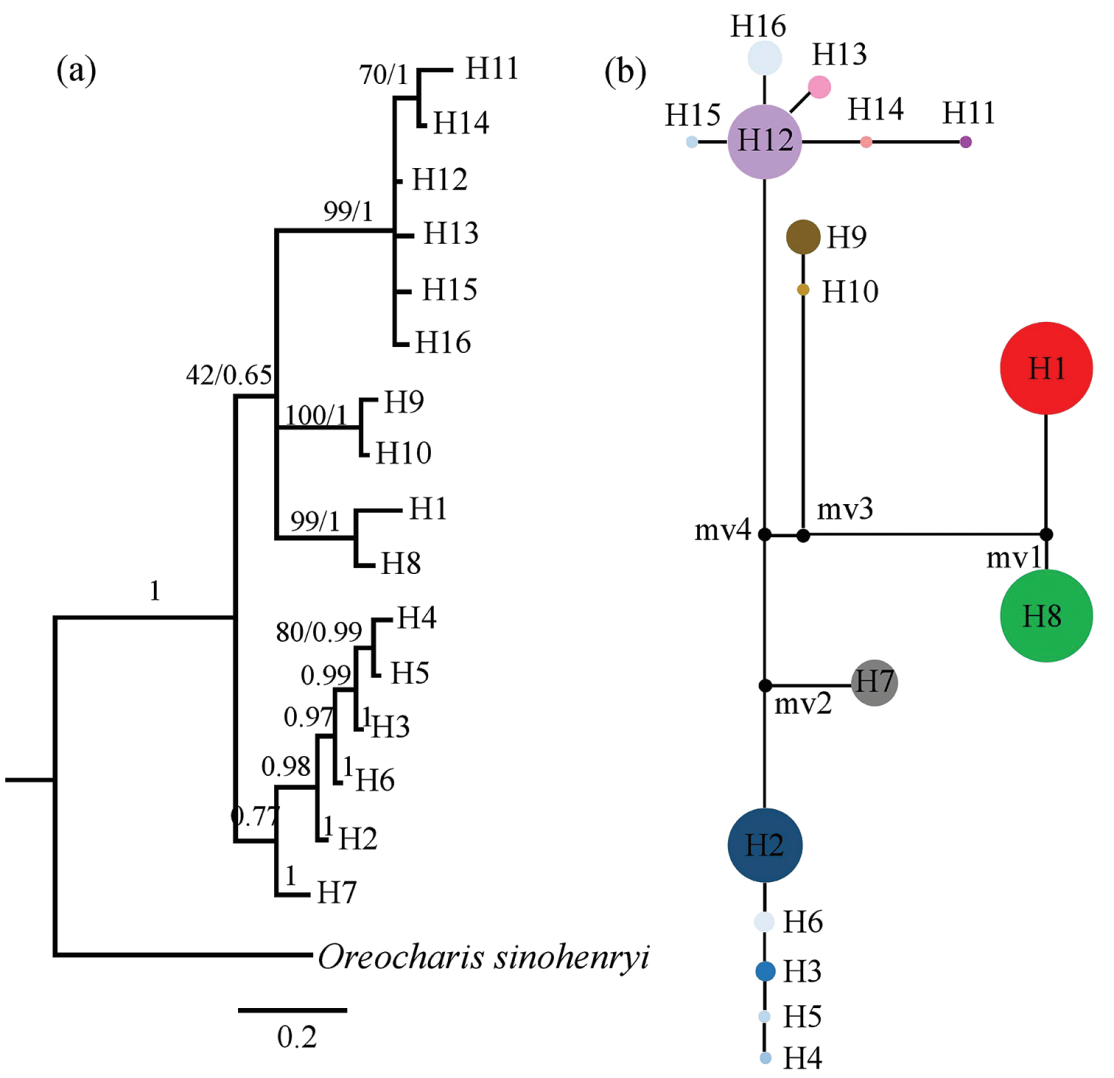
Bayesian Inference tree (**a**) using MrBayes and network (**b**) showing the genetic relationships amongst the observed ITS nucleotypes of Hainan *Oreocharis* populations. Numbers on branches indicate the bootstrap values for MP/MB and posterior probability. The relative sizes of the circles in the network are proportional to the nucleotype frequencies and missing nucleotypes are represented by a small black spot.

**Figure 3.**
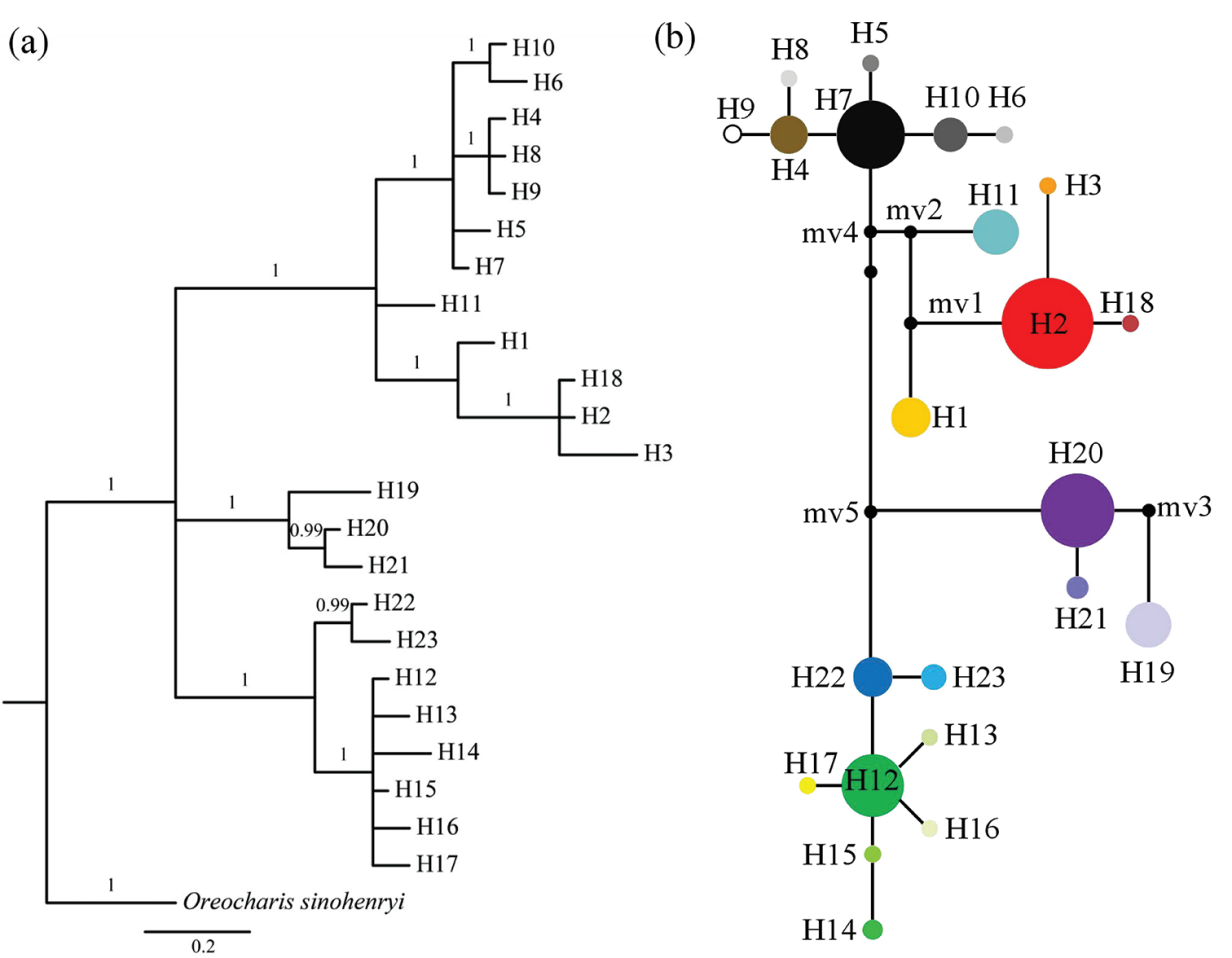
Bayesian Inference tree (**a**) and network (**b**) of *trn*L-F and *ycf*1b haplotypes of *Oreocharis* populations in Hainan Island. Posterior probabilities are given above branches. The relative sizes of the circles in the network are proportional to the haplotype frequencies and missing haplotypes are represented by a small black spot.

Both phylogenetic trees, based on ITS nucleotypes and cpDNA haplotypes, indicated that nucleotypes/haplotypes can be separated into three main groups with strong bayesian probabilities (> 0.95) (Figs 2, 3). The nucleotype/haplotype network of nuclear DNA and cpDNA was concordant with the phylogenetic relationship, which presented three centrally located nodes, representing possible ancestral haplotypes with a high frequency (Figs 2b, 3b). The rest of the haplotypes were connected to the central haplotypes by one to four steps in a star-like network. In the network of nuclear DNA, nucleotypes H1 and H8 occurred at the highest frequency, indicating they probably are ancestral nucleotypes of *O. dasyantha*. In the network of cpDNA, haplotypes H12 may be the ancestral haplotypes of *O. flavida* since it was at the centrally located nodes with highest occupied frequency.

### Neighbour-joining (NJ) tree and Population structure

The results of the NJ tree, based on nrDNA and cpDNA, suggested 12 populations were clearly clustered into three major groups, which well corresponded to the three defined *Oreocharis* taxa in Hainan Island, i.e. *O. dasyantha* (includes O. dasyantha var. ferruginosa), *O. flavida* and *Oreocharis* sp. Additionally, the analyses also presented a close relationship between *O. flavida* and *Oreocharis* sp., then with *O. dasyantha*.

Although the signal was stronger for cpDNA (*R*xy = 0.473, *P* < 0.001) than for nuclear DNA (*R*xy = 0.257, *P* < 0.001), the relationship between genetic and geographical distance for 12 populations was significant both in nuclear DNA and cpDNA (Appendix 3).

### Ordinations of morphological traits

According to the floral syndromes, the Principal Component Analysis of 16 floral characters of Hainan *Oreocharis* populations can be divided into three clusters (Fig. 5): (1) tubular, zygomorphic flowers with yellow tube but orange limbs, monomorphic stamens, pollen presentation separated (populations DW, DE, FT, NG, HM, CH and JF of *O. dasyantha* and O. dasyantha var. ferruginosa); (2) campanulate, zygomorphic, orange flower with included stamen and stigma (populations WZA, WZB and QX of *O. flavida*); (3) thin tubular, zygomorphic yellow flower with included didynamous stamens (populations YG and LM of *Oreocharis* sp.). The corolla colour and corolla shapes may play a key role in ordinations of morphological traits with high values of 45.41% and 34.04%, followed by location of stigma and length of the corolla tube with the values 9.293% and 5.272%.

## Discussion

### Monophyly of the Hainan Oreocharis taxa

The phylogenetic tree showed that Hainan *Oreocharis* taxa are monophyly (Appendix 1), suggesting a single dispersal of *Oreocharis* into Hainan Island. The sister species to Hainan *Oreocharis* is *O. sinohenryi*, which is restricted to South China including Guangxi and Guangdong provinces. Hainan Island is only about 30 km from these provinces, thus such observed pattern can be simply explained by geographic relationships.

### Genetic diversity and structure

Most *Oreocharis* populations hold very low nucleotide and haplotype diversity (Table 1) and overall populations revealed a high level of genetic differentiation (Table 3) and a significant phylogeographical structure. The three groups, i.e. *O. flavida*, *O. dasyantha* (including the variety O. dasyantha var. ferruginosa) and an unknown species showed clear geographical isolations associated with three different river ranges. *O. dasyantha* are found mostly in the watershed of the Changhua River (the secondly largest river on the island), *O. flavida* distributes in Mt. Wuzhi and Mt. Qixian, the upper reaches of the Wanquan River. The unknown species is restricted in the upper reaches of the largest river on the island, i.e. Nandu River. We concluded that these three groups may have evolved and maintained largely through allopatric differentiations.

Mountains can also probably explain such observed pattern with geographic isolation of these groups. Almost all *Oreocharis* populations in Hainan Island were restricted in > 1000 m high-elevation mountains with massive humidity, such that the island-like habitat became fragmented caused by a deep and wide valley in the complicated mountains system, which resulted in blocking of gene flow of *Oreocharis* populations with weak seed dispersal ability even at the fine scale (Xing et al. 2018).

### Genetic differentiation and species delimitation

**Figure 4.**
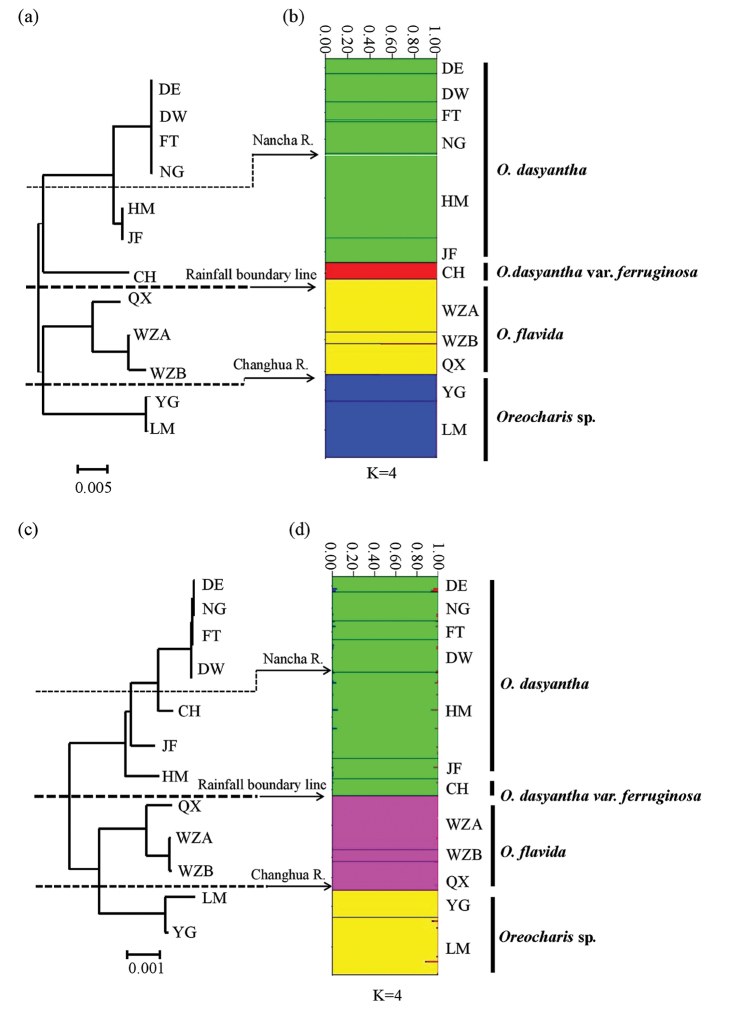
Neighbour-joining (NJ) tree based on ITS (**a**) and combined *trn*L-F and *ycf*1 (**c**) with the results of STRUCTURE, based on ITS (**b**) and combined *trn*L-F and *ycf*1 (**d**).

Li et al. (2019) found that geographic isolation by Changhua River is a driving force for the strong population differentiation in the Hainan-endemic *Primulina heterotricha* Merr. and *Metapetrocosmea peltata* (Merr. et Chun) W. T. Wang. Our results can also be explained by the isolation of Changhua River (Figs 1, 4), which indicated that Changhua River may play a key role in driving population divergence and speciation of the Hainan *Oreocharis* taxa (Xing et al. 2018; Li et al. 2019).

Secondly, the ‘sky island’ caused by high mountains may also cause such genetic differentiation for montane species (Palma-Silva et al. 2011; Tapper et al. 2014; Robin et al. 2015). Mountain tops in Hainan Island have tropical mountain cloud forests (> 1200 m) in Mt. Wuzhi, Mt. Yingge, Mt. Bawang, Mt. Jianfeng and Mt. Limu (Wang et al. 2016) which fragmented and restricted the island-like habitat of *Oreocharis* taxa. Alpine plant radiations have accelerated speciation with trait diversification (Sanderson 1998; Colin and Ruth 2006; Hughes and Atchison 2015) and, in general, these radiations are geared to be recent and rapid (Linder 2008). Almost all *Oreocharis* taxa in Hainan Island lived in high mountains except Population CH, which grew in a low-altitude habitat and held a distinct structure from other high-altitude populations of *O. dasyantha*, indicating the sky-island effect may drive population divergence and speciation.

**Figure 5.**
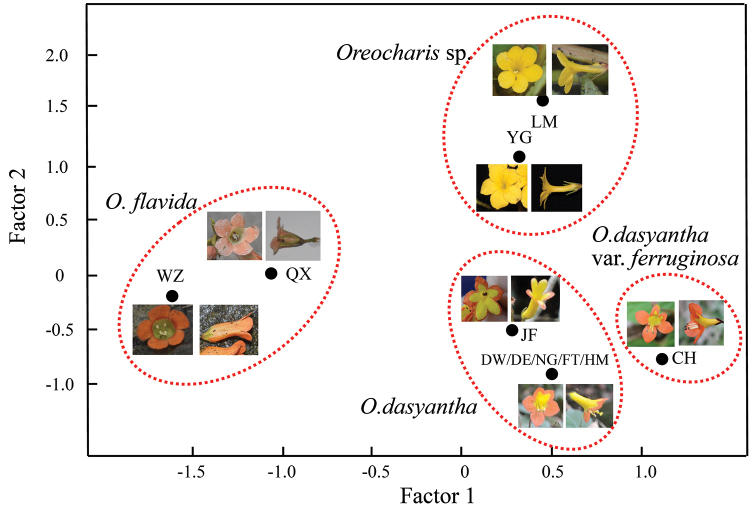
Principle Component Analysis of 16 morphological traits for the Hainan *Oreocharis* populations. Different clusters are shown in red circles.

According to morphological traits, all the 12 *Oreocharis* populations were also grouped into three clusters and corolla colour, shape and types are the main characters for distinguishing groups (Fig. 5; Ling et al. 2017). Such differences in floral syndromes further indicate the *Oreocharis* on Hainan Island should be recognised as three different lineages (species). Besides two species (includes one variety) currently recognised in Hainan Island, populations from Mt. Limu and Mt. Yingge should be treated as a new species or subspecies, which is still in need of further illumination.

## Figures and Tables

**Figure:**
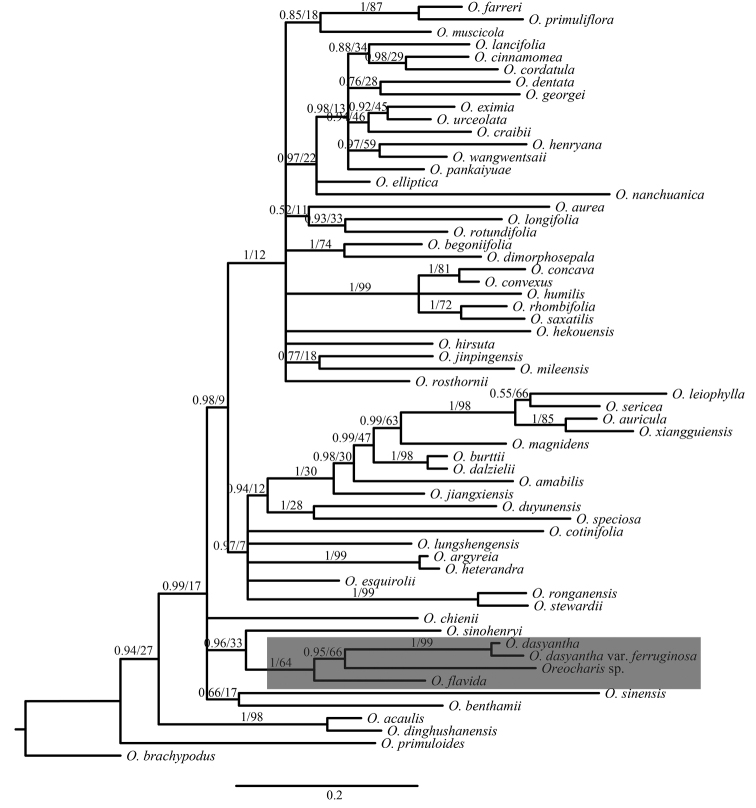
Molecular phylogeny for Hainan *Oreocharis* taxa and 57 *Oreocharis* species based on combined ITS1/2 and *trn*L-*trn*F, there was no significant incongruence based on the incongruence length different (ILD) test between the ITS1/2 and *trn*L-*trn*F. Bayesian posterior probability (PP) and ML bootstrap values (BS) are showed above branches. Hainan *Oreocharis* taxa were showed in grey.

**Figure:**
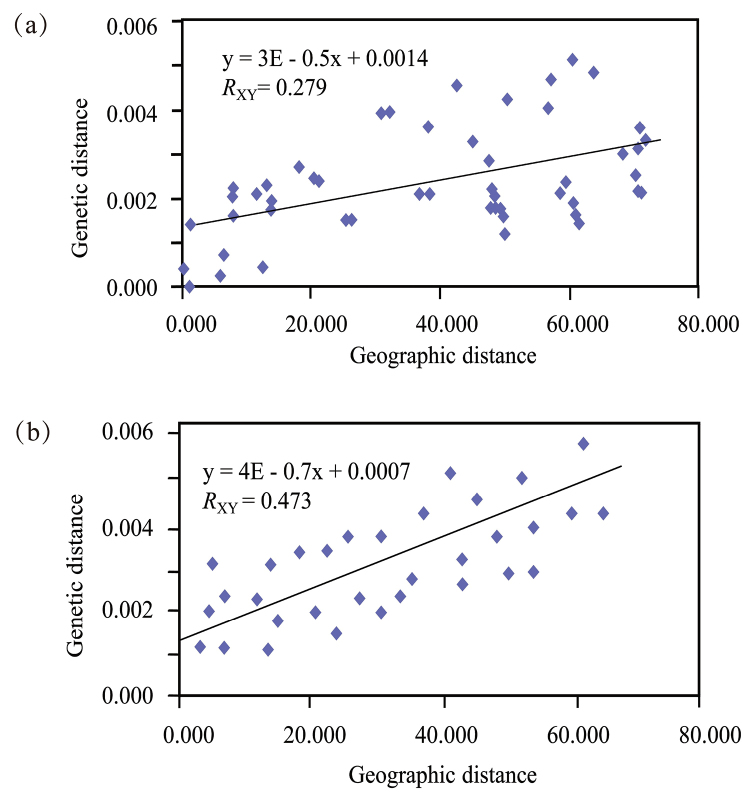
The results graph of the relationship between genetic and geographical distance for 12 populations based on the (**a**) ITS1/2 and (**b**) cpDNA

## Appendix 2

**Table:** List of Hainan *Oreocharis* taxa and 57 *Oreocharis* species used in the phylogenetic analysis, including respective Genbank accession and voucher numbers.

Species	*trn*L-*trn*F	ITS1/2	Voucher number
*Oreocharis acaulis* (Merr.) Mich.Möller & A.Weber	HQ633012	HQ632916	*M.Möller MMO 09-1605*
*Oreocharis amabilis* Dunn	KM232654.1	KJ475433.1	*Carles 587*
*Oreocharis argyreia* Chun ex Pan	HQ632919.1	HQ633015.1	*M.Möller MMO 07-1131*
*Oreocharis aurea* Dunn	KM062914.1	KM063154.1	*M.Möller MMO 06-980*
*Oreocharis auricula* (S.Moore) C.B.Clarke	FJ501482.1	DQ912664.1	*M.Möller MMO 03-304*
*Oreocharis begoniifolia* (H.W.Li) Mich.Möller & A.Weber	KM062926.1	KM063166.1	*M.Möller MMO 08-1221*
*Oreocharis benthamii* C.B.Clarke	JF697584.1	JF697572.1	*M.Möller MMO 08-1317*
*Oreocharis brachypodus* J.M. Li & Z.M.Li	KR476564.1	KR337019.1	*Jia-Mei Li 2304*
*Oreocharis burttii* (W.T.Wang) Mich.Möller & A.Weber	JF697582.1	JF697570.1	*F.Wen 2010-05*
*Oreocharis chienii* (Chun) Mich.Möller & A.Weber	KM062908.1	KM063148.1	*JXU0008123*
*Oreocharis cinnamomea* Anthony	KM062921.1	KM063161.1	*PE-02053073*
*Oreocharis concava* (Craib) Mich.Möller & A.Weber	KM062930.1	KM063170.1	*PE-02053062*
*Oreocharis convexa* (Craib) Mich.Möller & A.Weber	FJ501337.1	FJ501506.1	*M.Möller MMO 01-176*
*Oreocharis cordatula* (Craib) Pellegrin	KM062922.1	KM063162.1	*PE-02053432*
*Oreocharis cotinifolia* (W.T.Wang) Mich.Möller & A.Weber	HQ632914	HQ633010	*Q.M.Chuan 01*
*Oreocharis craibii* Mich.Möller & A.Weber	HQ632921	HQ633017	*M.Möller MMO 07-1072*
*Oreocharis dalzielii* (W.W.Sm.) Mich.Möller & A.Weber	JF697571	JF69783	*F.Wen 2010-06*
*Oreocharis dentata* A.L.Weitzman & L.E.Skog	KM062916.1	KM063156.1	*GH00353683*
*Oreocharis dimorphosepala* (W.H. Chen & Y.M. Shui) Mich.Möller	KM062925.1	KM063165.1	*Y. M.Shui & al. 85333*
*Oreocharis dinghushanensis* (W.T.Wang) Mich.Möller & A.Weber	GU350643	GU350675	*Lin Q.B. LQB06-01*
*Oreocharis duyunensis* Z.Y. Li, X.G. Xiang &Z.Y. Guo	MG722858.1	MG722856.1	*PE-02114626*
*Oreocharis elliptica* Anthony	KM063155.1	KM062915.1	*CDBI0130369*
*Oreocharis esquirolii* Léveillé	HQ633011	HQ632915	*D.W.Zhang 723*
*Oreocharis eximia* (Chun ex K.Y.Pan) Mich.Möller & A.Weber	KM062919.1	KM063159.1	*PE-02052811*
*Oreocharis farreri* (Craib) Mich.Möller & A.Weber	JF697585	JF697573	*Zhou Ping ZP 2010-020*
*Oreocharis georgei* Anthony	KM062917.1	KM063157.1	*PE-02053075*
*Oreocharis hekouensis* (Y.M.Shui & W.H.Chen) Mich.Möller & A.Weber	KM062934.1	KM063174.1	*KUN-1219106*
*Oreocharis henryana* Oliver	JF697586.1	JF697574.1	*CSH0017984*
*Oreocharis heterandra* D.Fang & D.H.Qin	KM232655.1	KJ475432.1	*PE-02052999*
*Oreocharis hirsuta* Barnett	KM062913.1	KM063153.1	*Put 3428*
*Oreocharis humilis* (W.T.Wang) Mich.Möller & A.Weber	GU350633	GU350665	*Liang R.H.SC-YB*
*Oreocharis jiangxiensis* (W.T.Wang) Mich.Möller & A.Weber	HQ633029	HQ632933	*M.Möller MMO 09-1451*
*Oreocharis jinpingensis* W. H. Chen & Y. M.	KM062923.1	KM063163.1	*Y.M. Shui et al. 91309*
*Oreocharis lancifolia* (Franch.) Mich.Möller & A.Weber	HQ632924	HQ633020	*M.Möller and P.Zhou MMO 09-1624*
*Oreocharis leiophylla* Wang	GU350676	GU350644	*Zhou X.R. ZXR-05-01*
*Oreocharis longifolia* (Craib) Mich.Möller & A.Weber	HQ632934	HQ633030	*M.Möller MMO 08-1239*
*Oreocharis lungshengensis* (W.T.Wang) Mich.Möller & A.Weber	HQ632917	HQ633013	*M.Möller MMO 06-916*
*Oreocharis magnidens* Chun ex Pan	HQ632930.1	HQ633026.1	*PE-02052989*
*Oreocharis mileensis* (W.T.Wang) Mich.Möller & A.Weber	KM063145.1	KM063182.1	*KUN-1385472*
*Oreocharis muscicola* (Craib) Mich.Möller & A.Weber	DQ912665	FJ501548	*Kew (1995-2229)*
*Oreocharis nanchuanica* (K.Y.Pan & Z.Y.Liu) Mich.Möller & A.Weber	KM062924.1	KM063164.1	*KUN-1385365*
*Oreocharis pankaiyuae* Mich.Möller & A.Weber	HQ632925	HQ633021	*PE-02053064*
*Oreocharis primuliflora* (Batalin) Mich.Möller & A.Weber	HQ633019	HQ932923	*PE-02053071*
*Oreocharis primuloides* (Miq.) Benth. & Hook.f. ex Clarke	FJ501546.1	FJ501364.1	*PE-01270488*
*Oreocharis rhombifolia* (K.Y.Pan) Mich.Möller & A.Weber	GU350632	GU350664	*PE-02053532*
*Oreocharis ronganensis* (K.Y.Pan) Mich.Möller & A.Weber	HQ633023	HQ632927	*PE-00030693*
*Oreocharis rosthornii* (Diels) Mich.Möller & A.Weber	KM062928.1	KM063168.1	*ZY0001346*
*Oreocharis rotundifolia* Pan	KM062911.1	KM063151.1	*PE-00030861*
*Oreocharis saxatilis* (Hemsl.) Mich.Möller & A.Weber	KM062932.1	KM063172.1	*JIU05295*
*Oreocharis sericea* Léveillé	KM232656.1	KJ475407.1	*CSFI059560*
*Oreocharis sinensis* (Oliv.) Mich.Möller & A.Weber	HQ632912	HQ633008	*IBSC-0548658*
*Oreocharis sinohenryi* (Chun) Mich.Möller & A.Weber	HQ632913.1	HQ633009.1	*M.Möller MMO 07-1150*
*Oreocharis speciosa* (Hemsl.) Mich.Möller & W.H. Chen	KM062909.1	KM063149.1	*K000858093*
*Oreocharis stewardii* (Chun) Mich.Möller & A.Weber	HQ632926	HQ633022	*M.Möller MMO 06-917*
*Oreocharis urceolata* (K.Y.Pan) Mich.Möller & A.Weber	KM062920.1	KM063160.1	*M.Möller MMO 09-1633*
*Oreocharis wangwentsaii* Mich.Möller & A.Weber	GU350658	GU350689	*Liang R.H.YN-Qj*
*Oreocharis xiangguiensis* W.T.Wang & K.Y.Pan	HQ632932.1	HQ633028.1	*JIU04686*
*Oreocharis dasyantha* Chun	MK587993	MK587954	*S.Jun Ling 20181124-02*
*Oreocharis flavida* Merr.	MK587990	MK587947	*S.Jun Ling 20181126-01*
Oreocharis dasyantha Chun var. ferruginosa K.Y. Pan	MK587992	MK587956	*S.Jun Ling 20181124-05*
*Oreocharis* sp.	MK587948	MK587987	*S.Jun Ling 20181205-01*

## Appendix 4

**Table:** Floral phenotypes in 16 characters of *Oreocharis* taxa in Hainan Island.

	DW	DE	FT	NG	HM	JF	CH	WZA	WZB	QX	YG	LM	Total Variance Explained
corolla color, coded as (0) yellow with orange, (1) yellow, (2) orange	0	0	0	0	0	0	0	2	2	2	1	1	45.406%
corolla shape and type, coded as (0) conical, (1) thin tubular, (2) campanulate	0	0	0	0	0	1	0	2	2	2	1	1	34.040%
corolla size, coded as (0) <1.49 cm, (1) 1.5 cm<-<1.99 cm, (2) >2.0 cm	1	1	1	1	1	1	1	1	1	0	2	2	0
corolla mouse width, coded as (0) <0.5 cm, (1) >0.5cm	1	1	1	1	1	0	1	1	1	1	0	0	0
length of tube, coded as (0) <0.99 cm, (1) 1 cm <-<1.49 cm, (2) >1.5 cm	2	2	2	2	2	2	2	1	1	0	1	1	5.272%
length of sepal, coded as (0)short, (1) long	0	0	0	0	0	0	0	0	0	1	0	0	0
number of petal, coded as (0) five, (1) six	0	0	0	0	0	0	0	0	0	0	0	1	0
location of stamens, coded as (0) included, (1) throat and (2) exerted	2	2	2	2	2	0	2	0	0	0	1	1	0
types of stamens, coded as (0) equal length, (1) didynamous stamens	0	0	0	0	0	0	1	0	0	0	1	1	0
pollen presentation, coded as (0) simultaneous, (1) separated	0	0	0	0	0	0	1	0	0	0	0	0	0
anther shape, coded as (0) oval shape, (1) horseshoe stage	0	0	0	0	0	0	0	1	1	1	1	1	0
hair on stamen, coded as (0) absent, (1) exist	1	1	1	1	1	1	0	1	1	1	0	0	0
location of stigma, coded as (0) included, (1) throat and (2) exerted	2	2	2	2	2	1	2	0	0	0	0	0	9.293%
number of stigma, coded as (0) one, (1) two	0	0	0	0	0	0	0	0	0	0	1	1	0
serration pf leaves edge, ceded as (0) absent, (1) exist	0	0	0	0	0	0	0	1	1	1	1	1	3.280%
leaf epiderrmal hair on abaxial side , ceded as (0) absent, (1) exist	1	1	1	1	1	1	1	0	0	0	1	1	0

Note: DW (Dongwu in Mt. Bawang), DE (Donger in Mt. Bawang), FT (Futou in Mt. Bawang), NG (Mt. Nangao), HM (Mt. Houmi), JF (Mt. Jianfeng), CH (Riverside at Chahe), WZA (Mt. Wuzhi A), WZB (Mt. Wuzhi B), QX (Mt. Qixian), YG (Mt. Yingge), LM (Mt. Limu)
